# Elevated red cell distribution width to platelet count ratio predicts poor prognosis in patients with breast cancer

**DOI:** 10.1038/s41598-019-40024-8

**Published:** 2019-02-28

**Authors:** Hideya Takeuchi, Miyuki Abe, Yohei Takumi, Takafumi Hashimoto, Michiyo Miyawaki, Tatsuro Okamoto, Kenji Sugio

**Affiliations:** Department of Thoracic and Breast Surgery, Oita University Faculty of Medicine, Oita, 879-5593 Japan

## Abstract

Red cell distribution width (RDW) to platelet ratio (RPR) is a prognosticator in acute pancreatitis and myocardial infarction; however, the prognostic values of RDW and RPR in breast cancer have not been studied. This retrospective analysis of 299 breast cancer patients investigated the association between RDW and RPR and clinicopathological characteristics and prognosis, compared to platelet distribution width to platelet count ratio (PDW/P) which is a known independent prognostic factor in patients with breast cancer. We found a significant correlation between RPR, and age and HER2 status. An elevated RPR significantly correlated with age and HER2 status. After a median follow-up duration of 48 months, tumour size, nuclear grade, PDW/P, and RPR were recgnized to be significantly associated with lower disease-free survival rates (tumour size: p < 0.01; nuclear grade, PDW/P, and RPR: p < 0.05) in univariate analysis. Tumour size and RPR were significant prognostic factors for lower disease-free survival rates, with hazard ratios of 4.31 (95% confidence interval: 1.76–10.53) (p < 0.01)] and 2.79 [95% confidence interval: 1.01–87.69) (p < 0.05)], respectively, in a multivariate analysis using the Cox proportional hazards model. This is the first study showing that an elevated RPR could independently predict poor prognosis in patients with breast carcinoma. Thus, RPR could be a novel biomarker for prognostic estimation.

## Introduction

Breast cancer is the most common malignant disease among women in Japan^1^. Despite the development of diagnostic and treatment modalities, breast cancer is one of the most frequent causes of cancer-related deaths^2^. For patients with breast cancer, prognostic estimation is crucial because it greatly impacts the selection of the most appropriate treatment. Several molecular diagnostic tests are applied to obtain reliable prognostic information in the United States and Europe, such as MammaPrint and Oncotype Dx. However, they are somewhat difficult to use in clinical situation in Japan, because the Japanese National Health Insurance could not follow their use owing to the high cost and regional unavailability of these kits^3^. Therefore, the liquid biopsy is used in the early detection of cancer; however, its clinical use is still limited due to its uncertain role and high cost^4^. Thus, there could be an urgent need to establish simple and low-cost prognostic biomarkers for breast cancer using routine haematological parameters of the complete blood count.

Red blood cell distribution width (RDW), an indicator of the variability in the sizes of circulating red blood cells, is routinely measured and automatically reported as part of the complete blood count, and has originally been used to differentiate the aetiology of anaemia for decades. Recently, RDW has gained substantial attention as an indicator of inflammation^5^, and a prognostic marker of cardiac and infectious disease^6,7^. Furthermore, accumulated evidence has also indicated that RDW could be a significant prognostic factor in esophageal^8^ and hepatocellular^9^ cancer. A novel index, RDW to platelet count ratio (RPR), has been shown to reflect the severity of inflammation and is used to predict fibrosis in chronic hepatitis^10^. Seretis *et al*. indicated that elevated RDW could be used as a supportive diagnostic tool to distinguish between benign and malignant breast tumours^11^. Seitanides *et al*. also revealed that RDW was significantly correlated with bone marrow metastatic spread in breast cancer patients^12^. However, to the best of our knowledge, no studies regarding the prognostic values of RDW and RPR in patients with breast cancer have been conducted. Recently, we demonstrated that platelet distribution width to platelet count ratio (PDW/P) was a significant prognostic factor in patients with breast cancer^13^. Hence, the purpose of the present study was to investigate the prognostic value of the RDW and RPR in breast cancer patients, compared with PDW/P.

## Methods

### Patients

This retrospective study comprised 299 patients with histologically confirmed breast cancer who underwent surgery at the Department of Thoracic and Breast Surgery, Oita University Faculty of Medicine between April 2006 and December 2017. As previously described in detail^13^, the exclusion criteria for our analysis included distant metastases at initial presentation, carcinoma *in situ*, bilateral breast carcinoma, and male breast carcinoma. Furthermore, we excluded patients with heart failure, on dialysis, and lacking the entire set of clinicopathological data in this study.

As previously described in detail^13^, adjuvant therapy was administered according to the recommendations of the St. Gallen panel^14^. A regular folow-up evaluation (3-month intervals during years 1–5 and at 6-month intervals during years 5–10 post-diagnosis) was performed. A radiological assessment (computed tomography and mammography), clinical examinations, and laboratory data analyses (carcinoembryonic antigen and carbohydrate antigen 15-3 levels) every 12 months during years 1–10 post-diagnosis were included as follow-up investigation.

### Clinicopathological characteristics

As previously described^13^, clinicopathological characteristics such as tumour size, nuclear grade, lymph node status, hormone receptor status, and human epidermal growth factor receptor 2 (HER2) status, were reviewed. Oestrogen receptor (ER) and progesterone receptor (PgR) statuses were evaluated via immunohistochemistry (IHC). Tumours with receptor expression scores above 0 were considered positive. HER2 status was assessed via IHC or fluorescence *in situ* hybridisation and was considered positive upon obtaining either an IHC score of 3 or at least a 2.2-fold stronger HER2 signal relative to the centromere enumerator probe 17 (CEP-17) signal in the tumour cells^15^.

### Measurement of RDW indices

Blood samples were collected via peripheral venous puncture before the initiation of any treatment modality. RDW and platelet count were measured routinely using an automatic nephelometer (XN-9000; Sysmex Corporation, Kobe, Japan) according to the manufacturer’s instructions. The RPR was calculated by dividing the RDW by the platelet count (×10^4^/μL). Both measurements were obtained from the same automated blood samples.

### Statistical analysis

EZR (Saitama Medical Center, Jichi Medical University, Saitama, Japan), a graphical user interface for R (the R Foundation for Statistical Computing, Vienna, Austria) was used for all statistical analyses. EZR is a modified version of the R Commander designed to include the statistical functions frequently used in biostatistics^16^. The student’s *t*-test was used for the comparison of variables between the two groups. The optimal cut-off values for PDW/P, RDW and RPR were determined by receiver operating characteristic (ROC) curve analysis by identifying the highest Youden index (sensitivity + specificity − 1).

The primary endpoint of the study was disease-free survival (DFS) defined as the interval between the dates of initial treatment and that of the first observation of disease relapse. Kaplan-Meier curve analysis and log-rank test were used to compare the survival of patients. Independent prognostic factors were identified via univariate analysis using a Cox proportional hazards model to identify any independent variables associated with DFS. Hazard ratios (HRs) estimated using Cox regression were reported as relative risks with their corresponding 95% confidence intervals (CIs). Multivariate Cox regression was performed for the parameters found to be significant in the univariate analysis. In this study, we opted to include factors with a p < 0.05 (instead of a p < 0.2) within the Cox regression model so as to be consistent and thus comparable with previous studies^17,18^. A p-value < 0.05 was considered statistically significant.

### Data collection and ethics compliance

The institutional ethics review board (the clinical research board of Oita University, institutional ID: 1407) approved for this retrospective study and granted use of the opt-out consent method. All medical data from the participants were anonymised and compiled. Because the study plan and choice to freely refuse participation were announced through the bulletin at the Oita University Faculty of Medicine, patients were recognized to have consented to the study if they did not refuse participation.

All procedures used in this study were performed in accordance with the tenets of the Declaration of Helsinki (1975) and its later amendments.

## Results

### Patients’ characteristics

The baseline characteristics of the patients are outlined in Table 1. The median age was 64.2 (range: 31–92) years at the time of diagnosis. With regards to PDW/P, RDW, and RPR, patients were divided into two groups according to the optimised cut-off values determined by ROC analysis.

**Table 1:** Baseline characteristics of the enrolled breast cancer patients.

Characteristics	No. (%)
**Age**	
<50	39 (13)
≥50	260 (87)
**Oestrogen receptor**	
Negative	80 (27)
Positive	219 (73)
**Tumour size (mm)**	
<20	201 (67)
≥20	98 (33)
**Nuclear grade**	
1	145 (48)
2, 3	154 (52)
**HER2**	
Negative	248 (83)
Positive	51 (17)
**Progesterone receptor**	
Negative	125 (42)
Positive	174 (48)
**Lymph node involvement**	
Negative	229 (77)
Positive	70 (23)
**PDW/P**	
<0.59	194 (65)
≥0.59	105 (35)
**RDW**	
<13.7	216 (72)
≥13.7	83 (28)
**RPR**	
<0.71	225 (75)
≥0.71	74 (25)

Abbreviations: No, number; HER2, human epidermal growth factor receptor 2; PDW/P, platelet distribution width to platelet count ratio; RDW, red cell distribution width; RPR, red cell distribution width to platelet count ratio.

ROC analysis showed that the optimal cut-off values for DFS were 0.59, 13.7 and 0.71 for the PDW/P, RDW and RPR, respectively (Table 2).

**Table 2:** Receiver operating characteristic curve analyses of red cell distribution width and red cell distribution width to platelet count ratio in breast cancer patients.

Characteristics	Optimal cut-off point	Specificity	Sensitivity	AUC (95%CI)
PDW/P	0.59	0.67	0.58	0.59 (0.47–0.72)
RDW	13.7	0.73	0.39	0.55 (0.44–0.67)
RPR	0.71	0.77	0.42	0.52 (0.38–0.65)

Abbreviations: AUC, area under the curve; PDW/P, platelet distribution width to platelet count ratio; RDW, red cell distribution width; RPR, red cell distribution width to platelet count ratio; CI, confidence interval.

The relationship between PDW/P, RDW & RPR and clinicopathological variables are shown in Table 3. The PDW/P was also found to be significantly correlated with age and HER2 status (p < 0.05).

**Table 3:** Association between platelet distribution width to platelet count ratio, red cell distribution and red cell distribution width to platelet count ratio and clinicopathological characteristics in breast cancer patients.

Variables	PDW/P			RDW		
	Average	SD	p-value	Average	SD	p-value
**Age (years)**						
<50	0.49	0.15	0.018	13.51	1.46	0.36
≥50	0.57	0.21		13.32	1.21	
**TS**						
<20	0.56	0.20	0.60	13.29	1.22	0.28
≥20	0.57	0.21		13.45	1.29	
**NG**						
1	0.57	0.20	0.37	13.34	1.31	0.98
2, 3	0.55	0.26		13.34	1.20	
**LN**						
(−)	0.56	0.19	0.70	13.28	1.18	0.09
(+)	0.57	0.26		13.56	1.43	
**ER**						
(−)	0.56	0.25	0.99	13.39	1.38	0.70
(+)	0.56	0.19		13.33	1.20	
**PgR**						
(−)	0.58	0.24	0.35	13.49	1.44	0.08
(+)	0.55	0.18		13.24	1.08	
**HER2**						
(−)	0.58	0.21	0.018	13.40	1.32	0.10
(+)	0.50	0.18		13.08	0.76	
**Variables**	**RPR**					
	**Average**	**SD**	**p-value**			
**Age (years)**						
<50	0.54	0.11	0.0026			
≥50	0.63	0.17				
**TS**						
<20	0.63	0.17	0.44			
≥20	0.61	0.17				
**NG**						
1	0.63	0.18	0.16			
2, 3	0.61	0.16				
**LN**						
(−)	0.62	0.17	0.46			
(+)	0.61	0.16				
**ER**						
(−)	0.60	0.17	0.29			
(+)	0.63	0.17				
**PgR**						
(−)	0.62	0.18	0.71			
(+)	0.62	0.16				
**HER2**						
(−)	0.63	0.17	0.019			
(+)	0.57	0.15				

Abbreviations: TS, tumour size; NG, nuclear grade; LN, lymph node involvement; ER, oestrogen receptor; PgR, progesterone receptor; HER2, human epidermal growth factor receptor 2; SD, standard deviation; p, p-value; PDW/P, platelet distribution width to platelet count ratio; RDW, red cell distribution width; RPR, red cell distribution width to platelet count ratio.

### Survival

After a median follow-up duration of 48 months, 26 patients (8.7%) had recurred. Univariate analysis revealed significant impacts of tumour size, nuclear grade, PDW/P, and RPR on DFS. Other variables were not found to be significantly correlated with DFS. On multivariate analysis, tumour size and RPR level were significantly correlated with poor prognosis for DFS, with HRs of 4.31 (95% CI: 1.76–10.53, p < 0.01) and 2.79 (95% CI: 1.01–7.69, p < 0.05), respectively (Table 4).

**Table 4:** Univariate and multivariate analyses of disease-free survival in breast cancer patients.

Characteristics	Univariate analysis		Multivariate analysis	
	Hazard ratio (95% CI)	p-value	Hazard ratio (95% CI)	p-value
**Age (years)**				
<50	1	0.08		
≥50	0.46 (0.19–1.09)			
**ER**				
Negative	1	0.29		
Positive	0.65 (0.29–1.45)			
**Tumour size**				
<20 mm	1	0.00057	1	0.0014
≥20 mm	4.14 (1.84–9.30)		4.31 (1.76–10.53)	
**NG**				
1	1	0.029	1	0.17
2, 3	2.62 (1.10–6.25)		1.93 (0.76–4.93)	
**HER2**				
Negative	1	0.71		
Positive	0.79 (0.24–2.65)			
**PgR**				
Negative	1	0.86		
Positive	0.94 (0.43–2.03)			
**LN**				
Negative	1	0.09		
Positive	1.97 (0.89–4.34)			
**PDW/P**				
<0.59	1		1	0.37
≥0.59	2.23 (1.02–4.85)	0.044	1.56 (0.59–4.13)	
**RDW**				
<13.7	1	0.26		
≥13.7	1.58 (0.72–3.50)			
**RPR**				
<0.71	1		1	0.048
≥0.71	2.21 (1.10–4.81)	0.046	2.79 (1.01–7.69)	

Abbreviations: NG, nuclear grade; LN, lymph node involvement; ER, oestrogen receptor; PgR, progesterone receptor; HER2, human epidermal growth factor receptor 2; PDW/P, platelet distribution width to platelet count ratio; RDW, red cell distribution width; RPR, red cell distribution width to platelet count ratio; CI, confidence interval.

**Figure 1:**
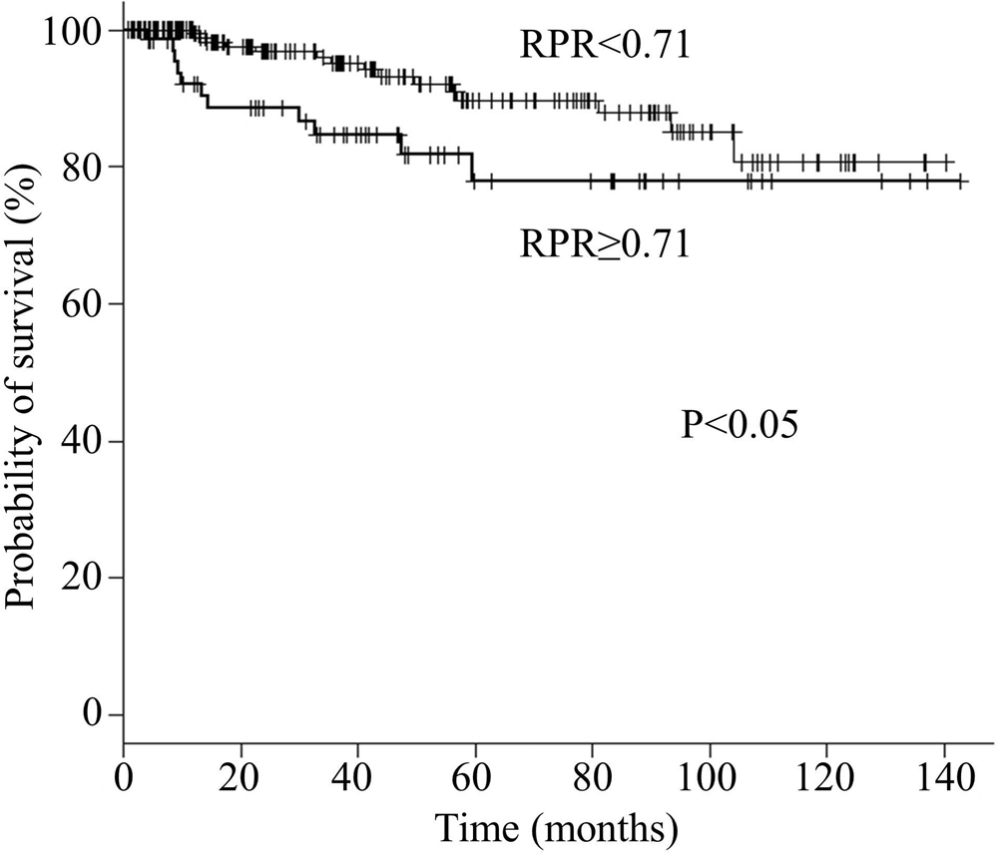
Kaplan-Meier analysis of disease-free survival stratified by the red cell distribution width to platelet count ratio in breast cancer patients.

The DFS rate in the elevated RPR group was significantly lower than in the low RPR group (5-year survival, 77.8% vs. 89.7%, respectively; p < 0.05) (Fig. 1).

## Discussion

To the best of our knowledge, this is the first study to demonstrate that the elevated RPR could be an independent risk factor for prognosis in breast cancer patients and is more powerful than PDW/P as a prognostic factor.

RPR was recognised as a strong predictor of the severity of fibrosis and cirrhosis in patients with chronic hepatitis^10^ and a valuable prognostic marker of inflammation in acute pancreatitis and myocardial infarction^19,20^. These results showed that RPR was regarded as an indicator of systemic inflammatory response. We have already demonstrated elevated levels of inflammatory markers, such as C-reactive protein and platelet to lymphocyte ratio to be related to poorer survival among breast cancer patients^21,22^. Therefore, it was biologically feasible that RPR was a reliable prognostic indicator in breast cancer, although there have been no reports regarding the value of RPR in the prognosis of malignant disease.

Although the specific mechanism underlying the poor prognosis of breast cancer patients with elevated RPR remains uncertain, it may be partially attributed to the inflammatory response and malnutrition. As tumour size increased, an extensive inflammatory reaction might be triggered and lead to an increase in the levels of circulating cytokines such as interleukin-6, tumour necrosis factor-α, and hepcidin^23,24^. These cytokines might suppress erythrocyte maturation and accelerate the entry of newer, larger reticulocytes into the peripheral circulation, thereby causing elevated RDW^25^. With regard to the association between RDW and nutrition status, malnutrition caused by deficiency of iron, folate and vitamin B12 owing to loss of appetite due to cancer could affect haematopoiesis, and thus amplify the heterogeneity of red blood cells, leading to an increase in RDW^26^. On the other hand, platelets are known to be associated with tumour growth and metastasis due to the release of various growth factors such as platelet-derived growth factor, vascular endothelial growth factor, and platelet factor 4^27^. However, the reason why the imbalances between RDW and platelet count could be a significant prognostic factor remains uncertain. Additional investigations are required to clarify the exact mechanisms bridging RPR and the survival of breast cancer patients.

The AUC of RPR was 0.52 which may be considered a small value. The AUC can be thought of as an indicator of overall ‘accuracy.’ A major practical fault of the AUC as an index of diagnostic performance is that it summarises the entire ROC curve, including regions that are frequently irrelevant to practical applications^28^. A large part of the area arose from the right side of the AUC where the high false positive fraction is of minimal clinical relevance^29^. Comparison of the AUCs between different screening or diagnostic tests may be meaningless^30^.

Some limitations of this study should be taken into account when interpreting the results. First, the analyses were performed on a small sample size, with a short-term follow-up period and single-centre. Secondly, there may inevitably be the potential for bias and inaccuracy in data collection as in most retrospectively studies. Third, the variability of the Youden index also depends on the clinical situation, because it could not differentiate between differences in sensitivity and specificity^30^. The cut-off point must, therefore, be judged in the context of the diagnostic situation to which the test is applied^31^. Furthermore, relevant laboratory data that can influence RPR levels, such as iron and vitamin B12 deficiency, were not collected and evaluated in this study.

Our study is the first to indicate that elevated preoperative RPR levels are indicative of unfavourable prognosis in patients with breast cancer. RPR, a cost-effective and easily calculated index almost universally available using two most common haematological parameters, can improve risk evaluation. However, our results are not conclusive and should not be considered for clinical practice unless further validation and feasibility studies have been completed.

## Data Availability

The datasets generated during and/or analysed during the current study are available from the corresponding author on reasonable request.
